# Awake ECMO for Mid‐Tracheal Obstruction: When a Tracheostomy Isn't Enough

**DOI:** 10.1002/ccr3.71948

**Published:** 2026-01-28

**Authors:** Jacob Beiriger, Maeher Grewal, Daniel Bestourous, Nilam D. Patel, Janani Vigneswaran, Vikas Sharma, Brian Mitzman, Jefferson H. Chambers, Hilary C. McCrary

**Affiliations:** ^1^ Department of Otolaryngology—Head and Neck Surgery University of Utah Salt Lake City Utah USA; ^2^ Department of Surgery Division of Cardiothoracic Surgery University of Utah Salt Lake City Utah USA; ^3^ Department of Internal Medicine, Division of Pulmonology University of Utah Salt Lake City Utah USA

**Keywords:** adenoid cystic carcinoma, airway management, extracorporeal membrane oxygenation, tracheal obstruction, tracheal surgery

## Abstract

Awake VV‐ECMO can be a life‐saving strategy for patients with near‐complete tracheal obstruction where tracheostomy and intubation are not feasible. Early multidisciplinary coordination enables safe airway control, tumor debulking, and stenting, facilitating both immediate stabilization and long‐term oncologic planning.

## 1 Introduction

Primary tracheal tumors are rare, comprising less than 0.1% of all malignancies, with adenoid cystic carcinoma (ACC) representing a small but challenging subset [1]. These tumors often present late and can cause progressive airway obstruction that may require urgent intervention. While tracheostomy is the traditional method for bypassing upper airway compromise, its utility diminishes in mid or distal tracheal lesions, where it may provoke bleeding or edema without relieving the obstruction.

In such anatomically constrained scenarios, extracorporeal membrane oxygenation (ECMO) has emerged as a valuable adjunct. Originally designed to support respiratory function, venovenous ECMO (VV‐ECMO) now plays a role in airway management, offering oxygenation and ventilation when intubation or tracheostomy are unsafe [2].

We present a rare case in which conventional airway interventions were not suitable in the setting of a near obstructive tracheal tumor. To our knowledge, this is one of the few reports describing the coordinated use of awake VV‐ECMO, interventional pulmonology techniques, and tracheal stenting for tracheal adenoid cystic carcinoma. By detailing the diagnostic, procedural, and collaborative elements involved in this patient's care, we aim to demonstrate a structured approach for managing similar airway emergencies and reinforce the expanding role of ECMO within the airway algorithm.

## 2 Case History/Examination

A 33‐year‐old otherwise healthy male presented with progressive dyspnea and sleep disturbance worsening over the past year. He reported positional shortness of breath and difficulty speaking in full sentences. On physical exam, he demonstrated biphasic stridor at rest but maintained oxygenation on room air (SpO_2_ 97%) and we completed expedited CT and office endoscopy under full monitoring.

Flexible transnasal tracheoscopy identified a friable, posterior mid‐to‐distal tracheal mass occupying nearly the entire lumen (Figure 2A). Only the in‐office bronchoscope could traverse the remaining patency. The distal trachea and carina appeared normal.

## 3 Differential Diagnosis, Investigations and Treatment

**FIGURE 1:**
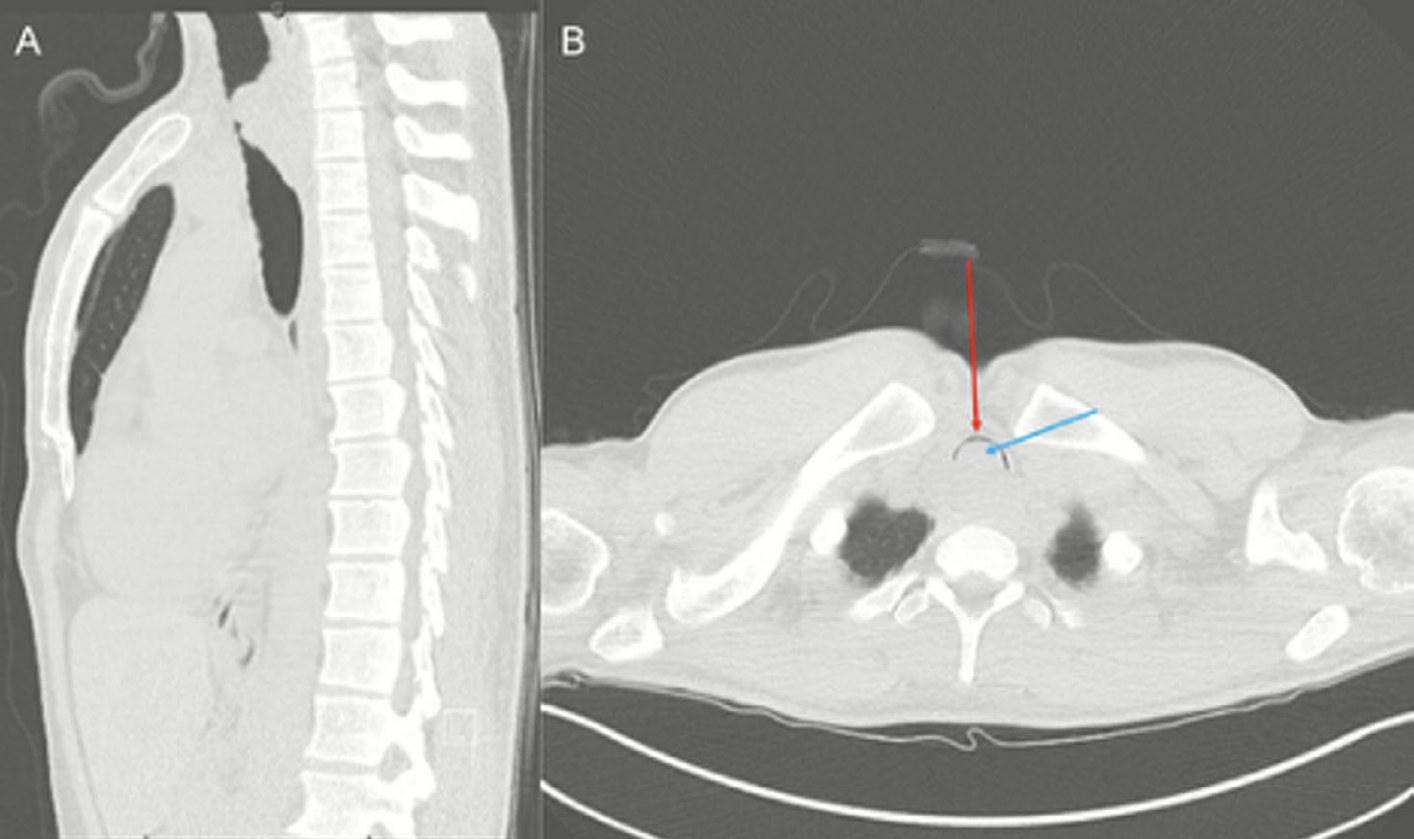
CT chest. (A) CT chest in the sagittal view shows near complete loss of tracheal lumen within the thorax. (B) Axial view of CT chest shows a sliver of patent airway in the anterior distal trachea reflected by a crescent of air (red arrow) and what is an obstructive soft tissue density occupying the remainder of the tracheal lumen (blue arrow).

Cross‐sectional imaging revealed a large and nearly occlusive tracheal mass (Figure 1). Office endoscopy confirmed a friable posterior mid‐to‐distal tracheal tumor occupying nearly the entire lumen. The differential diagnosis included primary tracheal malignancy, thyroid carcinoma with tracheal invasion, and benign tracheal tumors such as papillomatosis or granuloma.

Given the lesion's location and degree of obstruction, standard endotracheal intubation was not possible, and tracheostomy was considered unsafe due to the risk of airway loss and minimal expected benefit. After multidisciplinary consultation with anesthesia and cardiothoracic surgery, the patient was brought to the operating room (OR) for awake venovenous extracorporeal membrane oxygenation (VV‐ECMO) via femoral cannulation to secure oxygenation during induction and debulking. Once oxygenation was established, general anesthesia was induced.

**FIGURE 2:**
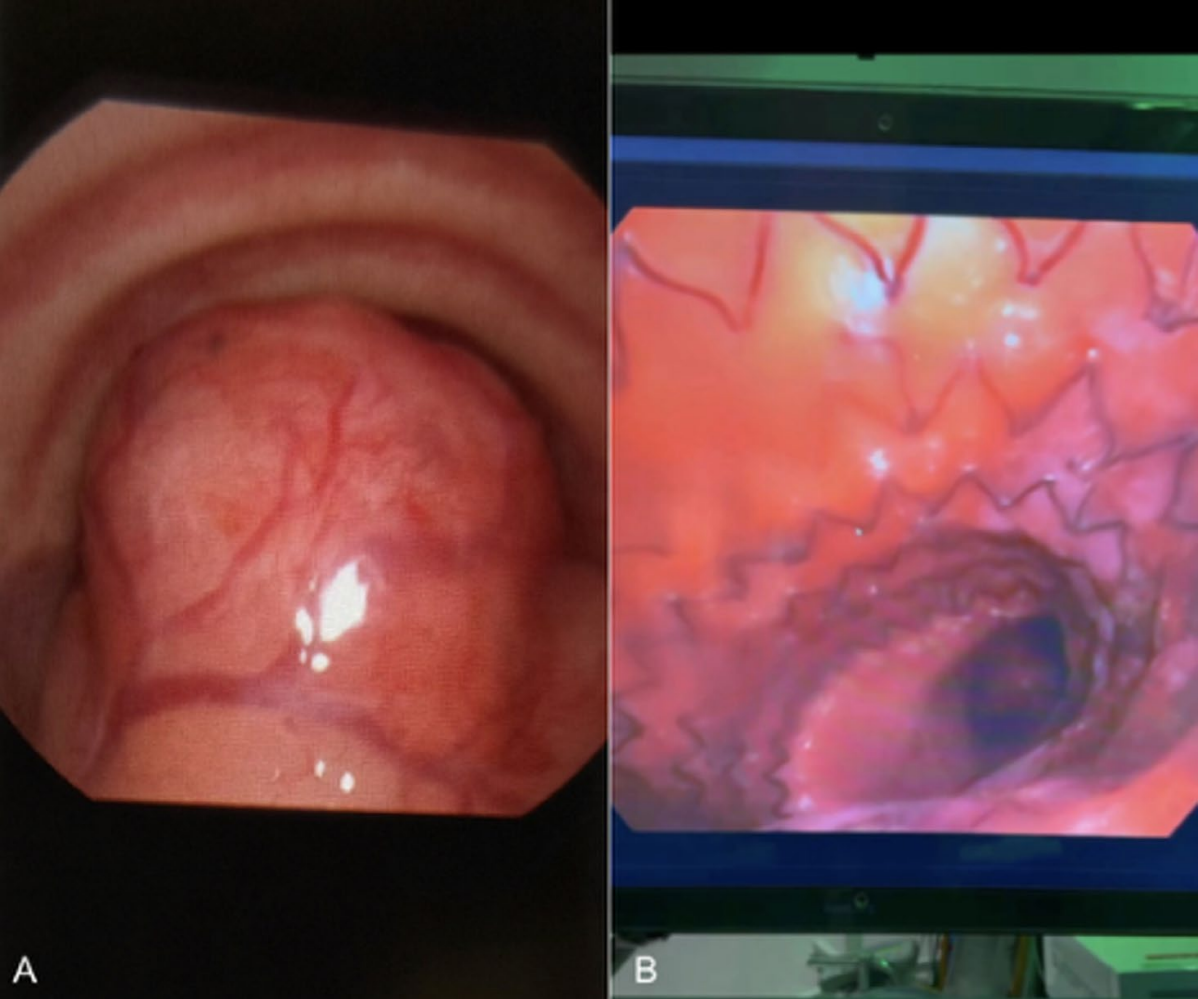
Endoscopic view. (A) In‐office flexible transnasal bronchoscopy reveals an exophytic, hyper‐vascular, posteriorly based tumor with normal tracheal rings seen anteriorly and no clear patency into the distal airway. (B) Intra‐operative photo of deployed intra‐luminal tracheal stent placed after tumor debulking prior to placement of endotracheal tube.

Interventional pulmonology performed endoscopic debulking using rigid bronchoscopy for exposure, followed by flexible bronchoscopy with electrocautery snare, cryotherapy, and forceps to remove tumor and obtain biopsy samples. Debulking was slow due to the tumor's firmness and poor response to cryoablation. After clearance, a temporary tracheal stent was placed to maintain airway patency (Figure 2B), and the patient was orotracheally intubated. Flexible esophagoscopy revealed no mucosal invasion but significant anterior wall mass effect.

## 4 Conclusion and Results

**FIGURE 3:**
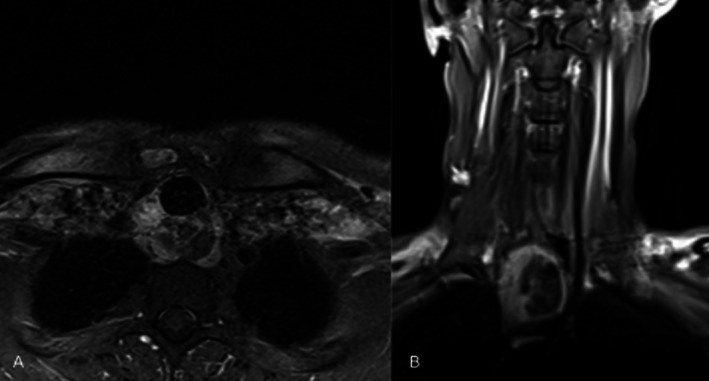
MRI neck and chest. (A) Axial T1 post contrast, fat suppressed MRI shows tumor encasing at least 180 degrees of the posterior tracheal wall, with mass effect onto esophagus causing leftward displacement without clear invasion. (B) Coronal T1 post contrast, fat suppressed MRI shows encapsulated tumor in the maximal craniocaudal direction encompassing the thoracic inlet.

The patient was monitored overnight in the cardiothoracic ICU, weaned from ECMO on postoperative day 1, and extubated the following day. ECMO was maintained overnight to allow for a controlled daytime wean and available airway teams and for post‐debulking edema or bleeding around the tracheal stent. Surgical pathology confirmed adenoid cystic carcinoma. MRI demonstrated a 5.5 cm craniocaudal lesion involving the posterior tracheal wall without overt esophageal invasion (Figure 3).

Following multidisciplinary tumor board review, the tumor was deemed unresectable due to submucosal extension and extensive tracheal involvement. The patient was referred for definitive chemoradiation and is undergoing molecular profiling for potential targeted therapy.

This study was approved by the University of Utah Institutional Review Board, and written informed consent was obtained.

## 5 Discussion

Primary malignancies of the trachea are exceptionally rare, and ACC represents a small but diagnostically elusive subset. These tumors often present with mild symptoms—dyspnea, cough, voice change—that are easily misattributed to asthma or other common conditions. As a result, many cases are diagnosed only after significant airway narrowing has occurred [1]. Histologically, ACC demonstrates perineural invasion and submucosal spread, contributing to a locally aggressive course and complicating surgical resection. Achieving negative margins is often not feasible due to the tumor's infiltrative behavior and proximity to critical airway structures.

In most airway emergencies tracheostomy is the cornerstone of surgical airway management. However, when obstruction lies distal to the typical tracheostomy site—as in this case near the thoracic inlet—its effectiveness is limited. Performing tracheostomy above a near‐occlusive lesion risks worsening edema, bleeding, or complete obstruction without improving airflow. Jet ventilation, while considered in some cases, is frequently insufficient when there is near‐total luminal compromise.

In these high‐risk situations, ECMO provides a life‐sustaining alternative. Awake VV‐ECMO facilitates oxygenation without instrumenting the airway, preserving spontaneous ventilation during cannulation and anesthetic induction. This technique has been increasingly adopted for airway rescue in tracheal tumors and complex central obstructions [2]. The 2025 American College of Chest Physicians guideline endorses ECMO for use in patients with central airway obstruction when ventilation cannot otherwise be established [3]. Similarly, the 2022 American Society of Anesthesiologists difficult airway algorithm lists ECMO as an option when conventional methods are likely to fail [4].

VV‐ECMO is typically sufficient for respiratory support for planned airway interventions while VA‐ECMO is reserved for emergency situations with hemodynamic compromise. Femoral cannulation with bilateral groin access is preferred when the neck is the operative field but has a higher recirculation risk than single‐cannula jugular access. Ongoing anticoagulation is generally unnecessary for short‐duration VV‐ECMO (especially when airway bleeding is a concern) and a low‐dose heparin bolus can be used to facilitate cannulation and reversed after initiation. VV‐ECMO flow rates approximating normal cardiac output (4–5 L/min) are typically adequate. ECMO can often be weaned immediately following airway stabilization and procedure completion in controlled settings. In this patient scenario ECMO was continued overnight to allow for a daytime wean with available airway teams and additionally monitor for post‐procedural edema or bleeding. Subsequent ventilator settings follow standard parameters unless the patient has underlying lung disease dictating otherwise.

Beyond establishing oxygenation, effective treatment of distal tracheal tumors requires technical tools that often lie outside the scope of routine otolaryngologic practice. Rigid endoscopy is limited in reach, particularly in patients with distorted or narrowed airways. Interventional pulmonology (IP) brings additional capabilities—rigid bronchoscopy, ablative or cryotherapy, and stenting—that are essential in these scenarios. In this case, IP was able to debulk the tumor using electrocautery, cryotherapy, and forceps, then place a temporary stent to preserve patency postoperatively. Such collaboration can be decisive in restoring the airway and enabling long‐term oncologic therapy. In the setting where ECMO is not available and the patient begins to decompensate, the rigid bronchoscope can be utilized to rapidly dilate and core out the obstructing lesion. While this may lead to bleeding, which needs to be dealt with after airway patency is regained, this is an important life‐saving maneuver that should be part of the treatment plan when necessary [5].

This case reinforces the need for early and deliberate multidisciplinary coordination. Otolaryngology, anesthesia, IP, and cardiothoracic surgery each contributed to a carefully choreographed sequence: awake VV‐ECMO cannulation, rigid and flexible endoscopic debulking, and stent placement. In situations where tracheostomy is not feasible, structured algorithms incorporating ECMO and IP techniques are vital. These strategies should not be improvised in crisis but rehearsed, institutionalized, and readily deployable in any center managing complex airway pathology.

## Author Contributions

**Jacob Beiriger:** conceptualization, data curation, investigation, writing – original draft, writing – review and editing. **Maeher Grewal:** data curation, investigation, writing – original draft, writing – review and editing. **Daniel Bestourous:** investigation, methodology, writing – review and editing. **Nilam D. Patel:** investigation, methodology, writing – review and editing. **Janani Vigneswaran:** investigation, methodology, writing – review and editing. **Vikas Sharma:** investigation, methodology, writing – review and editing. **Brian Mitzman:** investigation, methodology, writing – review and editing. **Jefferson H. Chambers:** project administration, supervision, writing – review and editing. **Hilary C. McCrary:** conceptualization, formal analysis, project administration, supervision, writing – review and editing.

## Funding

The authors have nothing to report.

## Consent

Written informed consent was obtained from the patient under the approval of the University of Utah Institutional Review Board for publication of this case report and associated images.

## Conflicts of Interest

The authors declare no conflicts of interest.

## Data Availability

The data that support the findings of this study are available from the corresponding author upon reasonable request.
